# Ultrasound-Guided Costoclavicular Brachial Plexus Block Using 0.5% Levobupivacaine Alone or With Dexamethasone as an Adjuvant for Upper Arm Surgical Anesthesia and Postoperative Analgesia: A Randomized Study

**DOI:** 10.7759/cureus.81658

**Published:** 2025-04-03

**Authors:** Kesa Ram, Mangilal Deganwa, Kalpana Verma, Avnish Bharadwaj, Vijay Mathur, Rajkumar K Saraswat

**Affiliations:** 1 Anesthesiology, Mahatma Gandhi Medical College and Hospital, Jaipur, IND; 2 Anesthesia and Critical Care, Mahatma Gandhi Medical College and Hospital, Jaipur, IND; 3 Anesthesiology, Mahatma Gandhi University of Medical Sciences and Technology, Jaipur, IND

**Keywords:** costoclavicular block, dexamethasone, levobupivacaine, regional anesthesia, upper arm surgeries

## Abstract

Background: Regional anesthesia techniques, such as brachial plexus blocks, are widely used for upper limb surgeries. The costoclavicular approach is a relatively new technique that targets the brachial plexus cords in a compact arrangement. Adjuvants like dexamethasone have been shown to enhance the efficacy of local anesthetics in various regional blocks, but studies regarding their effect in costoclavicular brachial plexus block (CCB) with levobupivacaine are limited.

Objective: The objective of this study is to compare the efficacy of 0.5% levobupivacaine alone versus 0.5% levobupivacaine with dexamethasone as an adjuvant for CCB in patients undergoing upper limb surgeries.

Methods: This prospective, randomized, observer-blinded controlled trial included 60 patients undergoing elective upper limb surgeries. Patients were randomly allocated into two groups: Group A (n = 30) received 18 mL of 0.5% levobupivacaine with 2 mL of normal saline, while Group B (n = 30) received 0.5% levobupivacaine 18 mL with dexamethasone 2 mL (8mg). The primary outcome was the onset of sensory blockade. Secondary outcomes included the onset of motor blockade, duration of sensory and motor blockade, time to first analgesic request, and complications.

Results: The dexamethasone group (Group B) demonstrated a significantly faster onset of both sensory (median 7 vs 9 minutes, p<0.01) and motor (median 11 vs 15.5 minutes, p<0.01) blockade. The duration of sensory (median 865 vs 496.5 minutes, p<0.01) and motor (median 840 vs 420 minutes, p<0.01) blockade was substantially prolonged in the dexamethasone group (Group B). Time to first analgesic request was significantly delayed in the dexamethasone group (median 847 vs 514 minutes, p<0.01). No significant complications were reported in either group.

Conclusion: The addition of dexamethasone to 0.5% levobupivacaine for CCB significantly enhances block efficacy, providing faster onset and prolonged duration, and extends postoperative analgesia compared to 0.5% levobupivacaine alone. This combination of levobupivacaine and dexamethasone can be a valuable option for optimizing regional anesthesia in upper limb surgeries.

## Introduction

Regional anesthesia techniques, such as brachial plexus blocks, have gained widespread acceptance in modern anesthesia practice due to their ability to provide effective analgesia, minimize systemic exposure to anesthetic agents, and facilitate early postoperative recovery [1]. Among the various approaches to brachial plexus blockade, the costoclavicular brachial plexus block (CCB) has emerged as a promising technique for upper limb surgeries, offering a reliable and efficient blockade of the brachial plexus [2].

The CCB, a modification of the infraclavicular brachial plexus block, targets the brachial plexus cords in the costoclavicular space, a relatively superficial and compact region located between the clavicle and the second rib [3]. In this space, the three cords of the brachial plexus (lateral, medial, and posterior) are closely clustered, facilitating a more consistent and compact distribution of the local anesthetic solution [2,4]. Compared to the classical infraclavicular approach, the costoclavicular technique has the advantage of targeting the brachial plexus at a more superficial level, potentially reducing the risk of complications associated with deeper needle insertion [2,3].

The costoclavicular approach has been gaining popularity due to its potential for rapid onset of action, reliable blockade, and phrenic nerve sparing blockade [3]. However, one of the limitations of regional anesthesia techniques is the relatively short duration of action of the local anesthetic agents used, necessitating the use of adjuvants to prolong the duration of analgesia and motor blockade [5].

Levobupivacaine, the S-enantiomer of bupivacaine, is a long-acting amide local anesthetic agent that has gained favor due to its reported lower cardiotoxic and neurotoxic potential compared to its racemic counterpart [6]. Despite its potential advantages, the duration of action of levobupivacaine may still be limited, prompting researchers to explore the use of adjuvants to enhance its effects.

Dexamethasone, a potent and highly selective glucocorticoid, has been extensively studied as an adjuvant to local anesthetic agents for various regional anesthesia techniques, including brachial plexus blocks [7]. Several mechanisms have been proposed to explain the potentiating effect of dexamethasone on local anesthetics. These include reducing the release of inflammatory mediators, inhibiting the ectopic discharge of neuronal membranes, and possibly inducing a degree of vasoconstriction, which may prolong the exposure of the nerve fibers to the local anesthetic [8,9].

Numerous studies have investigated the effects of adding dexamethasone as an adjuvant to local anesthetics for various regional anesthesia techniques, such as brachial plexus blocks, epidural anesthesia, and peripheral nerve blocks [10]. The addition of dexamethasone has been shown to prolong the duration of sensory and motor blockade, as well as postoperative analgesia, in a variety of regional anesthesia techniques [11,12]. However, the literature available on the use of dexamethasone as an adjuvant to levobupivacaine for the CCB is limited.

Most studies investigating the effects of dexamethasone as an adjuvant to local anesthetics have focused on other approaches to brachial plexus blockade, such as interscalene or axillary blocks, or have used different local anesthetic agents, such as bupivacaine or ropivacaine [13,14]. While these studies provide valuable insights into the potential benefits of using dexamethasone as an adjuvant, the costoclavicular approach offers a unique anatomical target and may yield different results compared to other approaches.

Furthermore, the pharmacokinetic and pharmacodynamic properties of levobupivacaine may interact differently with dexamethasone compared to other local anesthetic agents, necessitating specific investigations into the effects of this combination for the CCB [2,3,6].

This study aims to evaluate the effects of adding dexamethasone as an adjuvant to levobupivacaine for the CCB in terms of the onset and duration of sensory and motor blockade, as well as the duration of postoperative analgesia. By comparing the outcomes between 0.5% levobupivacaine alone and with dexamethasone, this study seeks to provide valuable insights into the potential benefits of using dexamethasone as an adjuvant for this specific regional anesthesia technique.

The findings of this study may have implications for clinical practice, guiding anesthesiologists in the selection of adjuvants and local anesthetic combinations for this specific regional anesthesia technique.

The primary objective included studying and comparing the onset of sensory and motor blockade following 0.5% levobupivacaine with and without dexamethasone used for CCB.

The secondary objectives included studying and comparing the duration of sensory, motor blockade and postoperative analgesia following 0.5% levobupivacaine with and without dexamethasone used for CCB, as well as examining the complications encountered with both of these techniques.

## Materials and methods

Study design and setting

This was a prospective, randomized, observer-blinded controlled trial conducted at the Department of Anaesthesia, Mahatma Gandhi Medical College, Jaipur from September 2022 to February 2024. The study protocol was approved by the Institutional Ethics Committee and registered with the Clinical Trials Registry of India (CTRI/2023/03/050642).

Sample size

Sixty consenting patients fulfilling criteria who underwent upper limb surgeries under ultrasound-guided CCB during the study period were included. Randomization was done using the computer box method.

Inclusion criteria

The study included patients scheduled for elective upper limb surgeries, including procedures involving the elbow, forearm, hand, and wrist. Patients of all genders were eligible to participate. The age range for participants was between 18 and 65 years. Only those classified as American Society of Anesthesiologists (ASA) physical status I or II were included in the study.

Exclusion criteria

The exclusion criteria for the study included any bleeding disorder or patients on anticoagulants, neurological deficits involving the brachial plexus, patients with an allergy to local anesthetics, local infection at the injection site, and patients on any sedatives or antipsychotics. Dropout patients were excluded from the study.

Procedure

After a thorough preanesthetic check-up the day before, patients were transferred to the operating room on the day of surgery. Upon arrival in the preoperative holding area, patients were randomly allocated to either Group A or Group B. Group A (n=30) received an ultrasound-guided CCB with 18 mL of 0.5% levobupivacaine with 2 mL of normal saline (total 20 mL). Group B (n=30) received an ultrasound-guided CCB with 18 mL of 0.5% levobupivacaine with 2 mL of dexamethasone (8 mg) (total 20 mL).

Block was given with a standard ASA protocol in the block area. The patients were placed in a supine position with the surgical limb abducted at an angle of 90 degrees. A transverse scan was performed using a linear probe (8-16 MHz) immediately below the midpoint of the clavicle and over the medial infraclavicular fossa. Maintaining the same position, the transducer was gently tilted cephalad to direct the ultrasound beam toward the costoclavicular space, defined as the space between the posterior surface of the clavicle and the second rib. The ultrasound image was optimized until all three cords of the brachial plexus were visualized lateral to the axillary artery. Using an in-plane technique and a lateral-to-medial direction, the block needle was advanced until its tip was located in the middle of the three cords. A total of 20 mL of study drug was injected between the medial posterior cord and the lateral cord.

**Figure 1:**
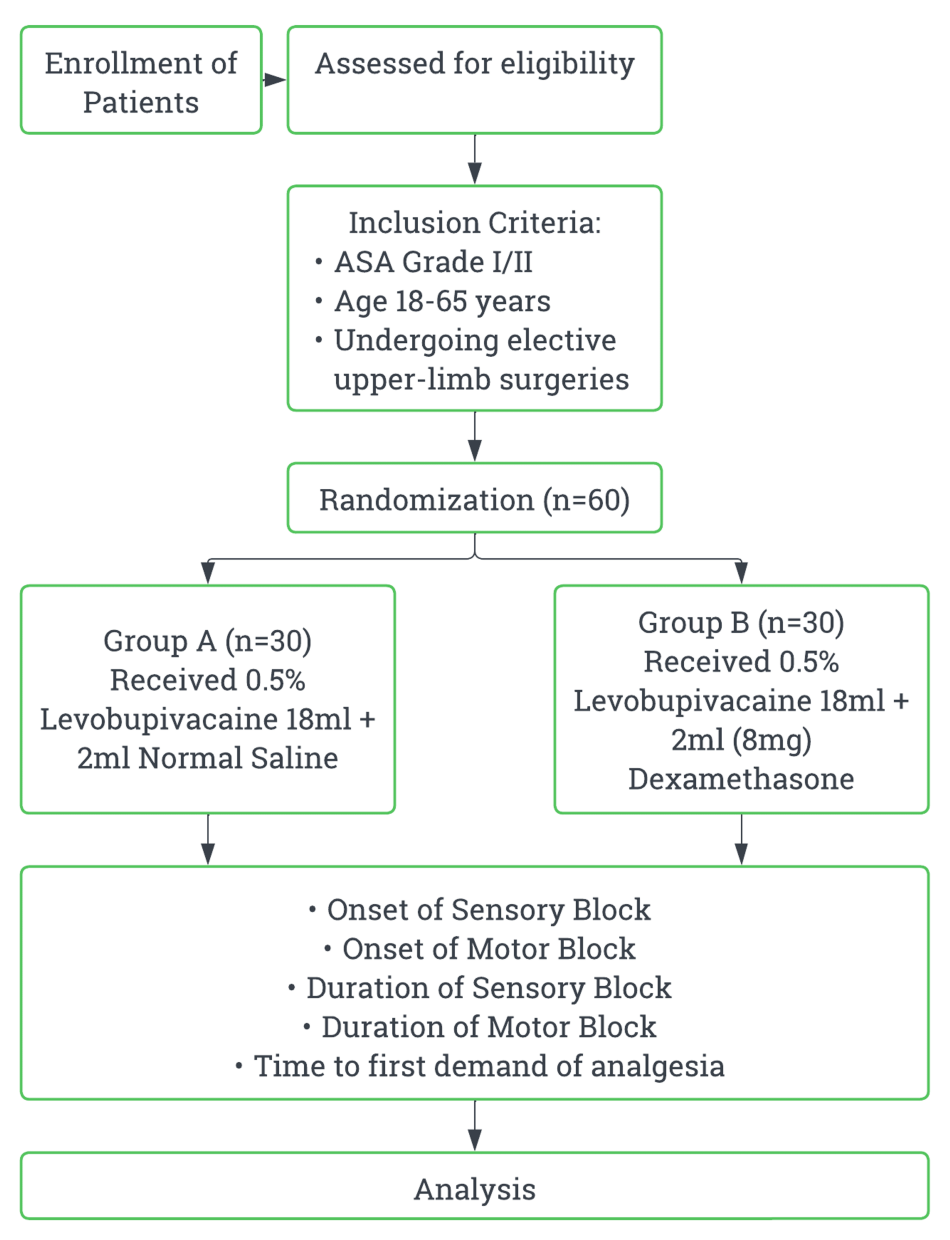
Study vignette Flowchart of the study process

After giving the block, the progress of the block was monitored by parameters like the onset and duration of sensory and motor block as well as the time to first demand of analgesia (Figure 1).

The pinprick test was used for sensory block assessment. Sensation was typically graded on a 3-point scale (0 = Normal sensation, 1 = Loss of sensation to pinprick (analgesia), 2 = Loss of sensation to touch (anesthesia)). Score 1 was used as an onset of sensory block. For duration, score 0 was used.

The modified Bromage scale for the upper limb was used for motor block assessment with a 5-point scale (0 = No motor block, 1 = Decrease in motor strength with ability to move the fingers only, 2 = Ability to flex the elbow and move the fingers, 3 = Inability to flex the elbow but able to move the fingers, 4 = Complete motor block). Score 2 was used as an onset of motor block. For duration, score 0 was used.

For rescue analgesia, injection tramadol 100 mg intravenous was given in 100 mL of normal saline when the VAS (visual analog score) was equal to or more than 4.

Analysis

Data were entered into Microsoft Excel (Microsoft® Corp., Redmond, WA) and statistical tests like the Mann-Whitney U test, independent t test, and Wilcoxon signed rank tests were applied based on the type of distribution of data and results were calculated. A p-value of < 0.05 was considered significant at 95% CI.

## Results

The age distribution of patients was comparable between Group A (levobupivacaine + normal saline) and Group B (levobupivacaine + dexamethasone). The Fisher's exact test revealed a p-value of 0.53, indicating that the age distribution did not differ significantly between the two groups. The minimum age of patients included in the study was 18 years, while the maximum age was 65 years (Table 1).

**Table 1:** Age & gender distribution of patients (N=60)

	Group A (n=30) Levobupivacaine + normal saline		Group B (n=30) Levobupivacaine + dexamethasone	
Age groups (years)	No.	%	No.	%
≤20	02	6.7	05	16.7
21-40	17	56.7	17	56.7
41-60	08	26.7	07	23.3
≥61	03	10	01	3.3
Fisher exact value=2.25, p-value=0.53				
Male	19	63.3	25	83.3
Female	11	36.7	05	16.7
χ^2 ^=3.06, df=1, p-value=0.08				

The gender distribution of patients was not completely balanced between the two groups, although the difference did not reach statistical significance. The chi-square test was used to assess the significance of the difference in gender distribution between the two groups. The corresponding p-value was 0.08, suggesting that the observed difference in gender distribution between the two groups is not statistically significant (Table 1).

The comparison of vital parameters between the two groups revealed no statistically significant differences in heart rate, systolic and diastolic blood pressure, respiratory rate, and temperature. The p-values for all these parameters were above 0.05, indicating that the groups were comparable in terms of these vital signs (Table 2).

**Table 2:** Comparison of vitals & investigations between both the groups (N=60)

Vitals & Investigations	Group A (n=30) Levobupivacaine + Normal saline		Group B (n=30) Levobupivacaine + dexamethasone		Test Statistics (Mann-Whitney U Test)	
	Median	IQR	Median	IQR	Z-value	p-value
Heart rate (bpm)	79.5	9	82	10	-1.53	0.12
Systolic blood pressure (mmHg)	126	12.25	127	11	-0.54	0.58
Diastolic blood pressure (mmHg)	79	9.75	80	10.25	-.0.49	0.61
Respiratory rate (per minute)	13.5	2	14	3	-1.76	0.07
Temperature (ºF)	98.6	0.2	98.6	0.3	-0.74	0.45

**Figure 2:**
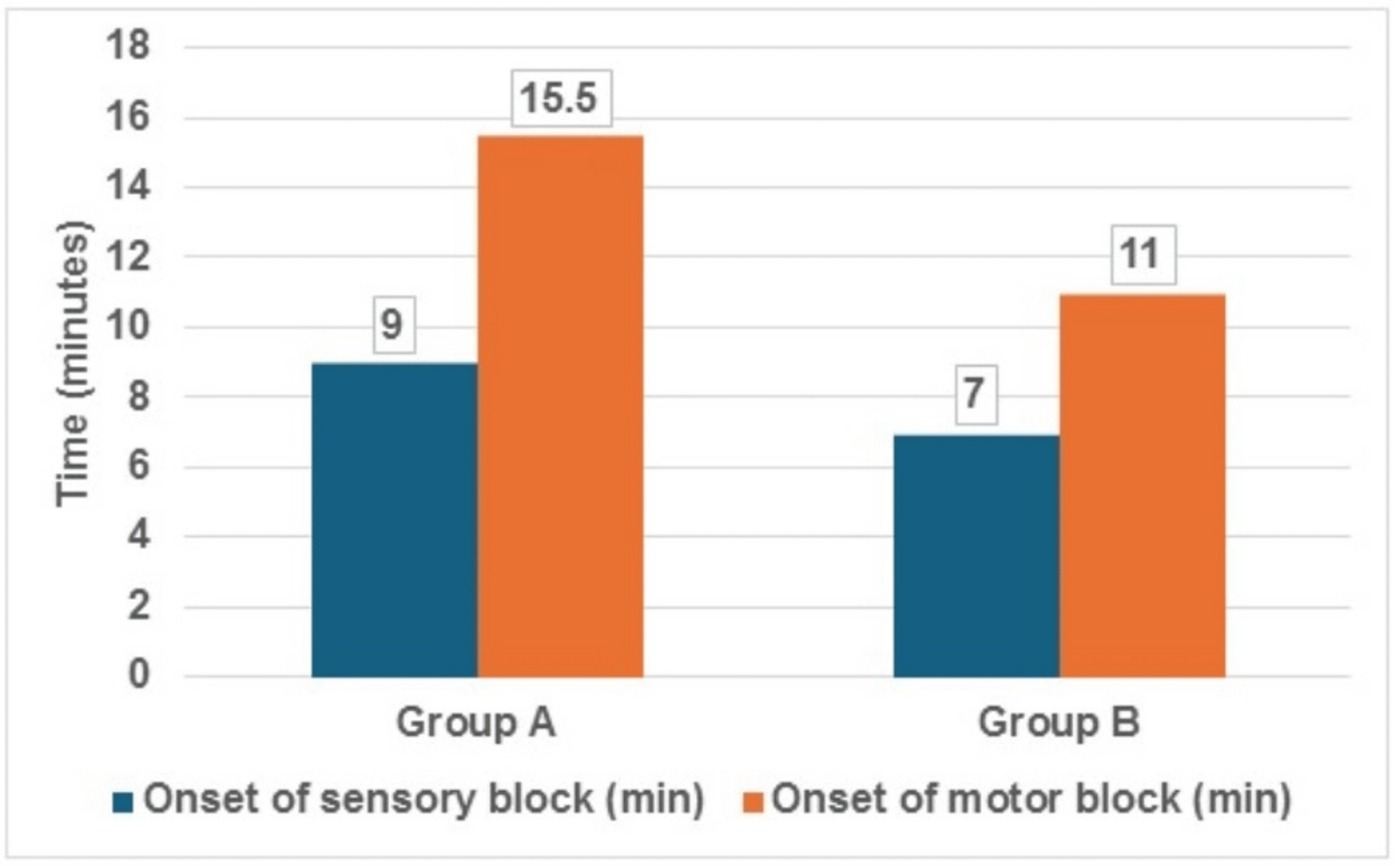
Comparison of sensory and motor block onset between both the groups (N=60)

**Figure 3:**
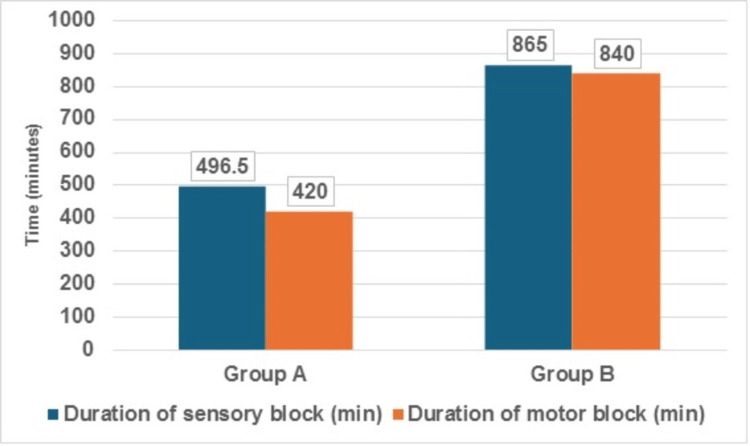
Comparison of sensory and motor block duration between both the groups (N=60)

**Figure 4:**
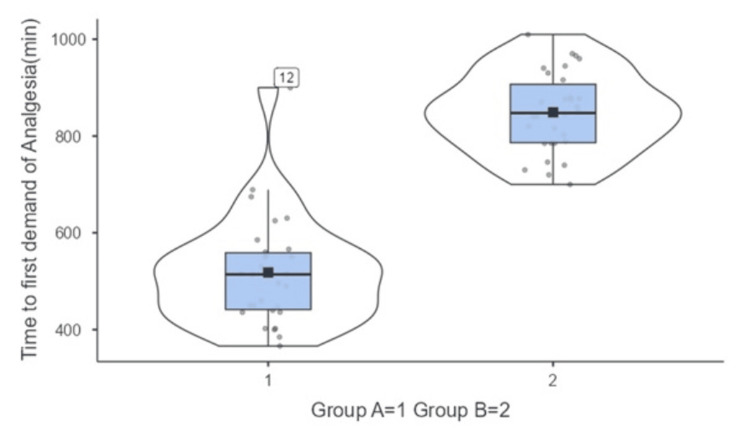
Comparison of time to first demand of analgesia (min) between both the groups (N=60)

Based on Table 3 and the accompanying Figures 2-4, it is evident that the addition of dexamethasone as an adjuvant to levobupivacaine for the CCB significantly impacted various block characteristics compared to levobupivacaine alone.

**Table 3:** Comparison of block characteristics between both the groups (N=60)

Block Characteristics	Group A (n=30) Levobupivacaine + Normal saline		Group B (n=30) Levobupivacaine + dexamethasone		Test Statistics (Mann-Whitney U Test)	
	Median	IQR	Median	IQR	Z-value	p-value
Onset of sensory block (min)	9	5	7	4.3	-2.63	<0.01
Onset of motor block (min)	15.5	6	11	4	-4.5	<0.01
Duration of sensory block (min)	496.5	128	865	136	-6.48	<0.01
Duration of motor block (min)	420	157	840	143	-6.65	<0.01
Time to first demand of Analgesia(min)	514	123	847	135	-6.32	<0.01

Onset of sensory and motor blockade

The median onset of sensory blockade was faster in Group B (levobupivacaine + dexamethasone) at seven minutes compared to nine minutes in Group A (levobupivacaine + normal saline), with a statistically significant difference (p < 0.01). Similarly, the median onset of motor blockade was more rapid in Group B (11 minutes) than in Group A (15.5 minutes), also with a statistically significant difference (p < 0.01). Figure 2 depicts these differences in the onset of sensory and motor blockade.

Duration of sensory and motor blockade

The addition of dexamethasone significantly prolonged the duration of both sensory and motor blockade. The median duration of sensory blockade was 865 minutes (14.4 hours) in Group B compared to 496.5 minutes (8.3 hours) in Group A (p < 0.01). Similarly, the median duration of motor blockade was substantially longer in Group B (840 minutes, or 14 hours) compared to Group A (420 minutes, or 7 hours) (p < 0.01). Figure 3 illustrates the marked differences in the durations of sensory and motor blockade between the two groups, with Group B exhibiting prolonged durations for both sensory and motor blockade.

Time to first demand of analgesia

Consistent with the prolonged duration of sensory blockade, the time to the first demand of analgesia was significantly longer in Group B (median of 847 minutes, or 14.1 hours) compared to Group A (median of 514 minutes, or 8.6 hours) (p < 0.01). Figure 4 visually represents this difference in the duration of postoperative analgesia between the two groups.

The interquartile ranges (IQRs) provided in the table indicate the variability in the data, with Group B generally exhibiting slightly higher IQRs than Group A, suggesting greater dispersion in the values for the dexamethasone group.

Overall, the addition of dexamethasone as an adjuvant to levobupivacaine for the CCB led to a faster onset of sensory and motor blockade, significantly prolonged durations of both sensory and motor blockade, and extended the duration of postoperative analgesia compared to levobupivacaine alone. These findings suggest that dexamethasone may be a valuable adjuvant for enhancing the efficacy and prolonging the effects of levobupivacaine in this regional anesthesia technique.

## Discussion

The present study aimed to compare the efficacy of 0.5% levobupivacaine alone versus 0.5% levobupivacaine with dexamethasone as an adjuvant for CCB in patients undergoing upper limb surgeries. The results demonstrated significant advantages in block characteristics with the addition of dexamethasone to levobupivacaine.

Block characteristics

Onset of Sensory and Motor Blockade

The study found a statistically significant difference in the onset of both sensory and motor blockade between the two groups which was our primary objective. The median onset time for sensory block was shorter in Group B (seven minutes) compared to Group A (nine minutes) (p<0.01). Similarly, the onset of motor blockade was faster in Group B (11 minutes) than in Group A (15.5 minutes) (p<0.01).

These findings are consistent with several previous studies that have reported a faster onset of action when dexamethasone is used as an adjuvant to local anesthetics in various regional anesthesia techniques. For instance, Choi et al. (2014) [7] conducted a meta-analysis of randomized controlled trials and found that perineural dexamethasone significantly hastened the onset of sensory and motor blockade in brachial plexus blocks. The mechanism behind this accelerated onset is not fully elucidated, but it may be related to the ability of dexamethasone to alter the function of potassium channels on nociceptive C-fibers, leading to hyperpolarization and reduced conduction of pain signals [15].

The faster onset of block in the dexamethasone group could have important clinical implications. A more rapid onset of anesthesia can lead to improved operating room efficiency, reduced preoperative anxiety for patients, and potentially earlier commencement of surgical procedures. However, it is worth noting that while statistically significant, the absolute difference in onset times between the groups was relatively small (two minutes for sensory block and 4.5 minutes for motor block). The clinical significance of these differences may depend on the specific context and requirements of individual surgical settings.

Duration of sensory and motor blockade

One of the most striking findings of this study was the marked prolongation of both sensory and motor blockade in the dexamethasone group which was our secondary objective. The median duration of sensory block was 865 minutes (14.4 hours) in Group B compared to 496.5 minutes (8.3 hours) in Group A (p<0.01). Similarly, the duration of motor block was substantially longer in Group B (840 minutes or 14 hours) compared to Group A (420 minutes or seven hours) (p<0.01).

These results align with a growing body of evidence supporting the block-prolonging effects of perineural dexamethasone. A systematic review and meta-analysis by Knezevic et al. (2015) [16] found that the addition of dexamethasone to local anesthetics for peripheral nerve blocks significantly extended the duration of analgesia and motor block. The magnitude of prolongation observed in our study (approximately 1.7-fold for sensory block and 2-fold for motor block) is comparable to or even greater than that reported in some previous studies.

The mechanism underlying this prolongation effect is likely multifactorial. Dexamethasone may act through both local and systemic mechanisms. Locally, it is thought to reduce nociceptive C-fiber activity and block transmission in unmyelinated C-fibers [17]. Systemically, it may exert anti-inflammatory effects that contribute to prolonged analgesia. Additionally, dexamethasone has been shown to induce vasoconstriction, which may slow the systemic absorption of the local anesthetic, thereby prolonging its local effect [18].

The extended duration of sensory blockade has significant implications for postoperative pain management. Prolonged analgesia can reduce the need for systemic opioids in the immediate postoperative period, potentially decreasing opioid-related side effects and improving patient satisfaction. However, the prolonged motor blockade observed in the dexamethasone group may be a double-edged sword. While it may be advantageous in certain surgical scenarios, extended motor blockade could potentially delay mobilization and rehabilitation in some patients.

Time to First Analgesic Request

Consistent with the prolonged sensory blockade, the time to first analgesic request was significantly longer in the dexamethasone group. This finding has important clinical implications for postoperative pain management and patient comfort.

The extended duration of analgesia observed in the dexamethasone group aligns with numerous previous studies. A meta-analysis by De Oliveira et al. (2014) [19] found that perineural dexamethasone significantly prolonged the duration of analgesia in brachial plexus blocks, with a mean difference of 473 minutes compared to control groups. Our study also showed a difference of 333 minutes between the groups, which could be attributed to factors such as the specific local anesthetic used (levobupivacaine), the block technique (costoclavicular approach), or the dose of dexamethasone employed.

The prolonged time to first analgesic request offers several potential benefits. It may reduce the overall analgesic requirements in the immediate postoperative period, potentially decreasing the risk of opioid-related adverse effects such as nausea, vomiting, and respiratory depression. This extended analgesia could also facilitate earlier mobilization and potentially earlier discharge in appropriate cases, aligning with enhanced recovery after surgery (ERAS) protocols [20].

However, it is important to note that while delayed onset of pain is generally beneficial, it does not necessarily equate to superior overall pain control throughout the entire postoperative period. Future studies could benefit from assessing pain scores at various time points and evaluating total analgesic consumption over the first 24-48 hours postoperatively to provide a more comprehensive picture of the analgesic efficacy.

Safety and side effects

The study did not report any significant complications or side effects in either group. This finding is reassuring and consistent with the established safety profile of both levobupivacaine and dexamethasone when used in regional anesthesia. Levobupivacaine, the S-enantiomer of bupivacaine, is known for its improved safety profile compared to racemic bupivacaine, particularly in terms of cardiovascular and central nervous system toxicity [21].

The safety of perineural dexamethasone has been a subject of much research and debate. While our study did not observe any adverse effects, it's important to note that the sample size may not have been sufficient to detect rare complications. Some concerns have been raised about the potential neurotoxicity of perineural steroids, but current evidence suggests that dexamethasone, at the doses commonly used in regional anesthesia, does not increase the risk of nerve injury [22]. A systematic review by Hussain et al. (2018) [23] concluded that perineural dexamethasone appears to be safe based on clinical and animal studies, with no reports of long-term neural toxicity in humans.

However, it is crucial to consider potential systemic effects of dexamethasone, particularly in susceptible populations. While a single dose is unlikely to cause significant issues in most patients, repeated doses or use in patients with diabetes, infection, or other contraindications to steroids should be approached with caution. Long-term follow-up studies would be valuable to further establish the safety profile of perineural dexamethasone.

Saraswat et al. (2024) in their study demonstrated that the CCB demonstrated superior preservation of diaphragmatic contractility and lesser deterioration of pulmonary function test compared to the supraclavicular approach while being equally effective. These findings highlight the potential benefits of the costoclavicular technique in minimizing diaphragmatic dysfunction and respiratory impairment, particularly in patients at risk for respiratory complications [24].

Limitations of the study

The study included 60 patients (30 per group). While this was sufficient to detect statistically significant differences in the primary and secondary outcomes, a larger sample size would increase the reliability and generalizability of the findings. The study was conducted at a single institution, which may limit its generalizability to other settings with different patient populations or practice patterns. The study used a fixed dose of dexamethasone (8 mg). Future studies could explore different doses to determine the optimal balance between efficacy and safety.

## Conclusions

This study provides strong evidence that dexamethasone, when used as an adjuvant to 0.5% levobupivacaine in CCB, significantly enhances block efficacy for upper limb surgeries. The findings have important clinical implications, suggesting that this combination can be a valuable option for optimizing regional anesthesia in these procedures. However, further research may be warranted to explore long-term effects, optimal dosing, and potential side effects in diverse patient populations and surgical contexts.
